# Prevalence of Probable REM Sleep Behavior Disorder in Essential Tremor

**DOI:** 10.5334/tohm.1214

**Published:** 2026-08-25

**Authors:** Peyman Petramfar, Steven Bellows

**Affiliations:** 1 Parkinson’s Disease Center and Movement Disorders Clinic, Department of Neurology, Baylor College of Medicine, Houston, Texas, USA; 2 Department of Neurology, University of Iowa Health Care, Iowa City, IA, USA

**Keywords:** Prevalence, RBD, REM Behavior Sleep Disorder, Essential Tremor, Parkinson’s Disease

## Abstract

**Background:**

Essential Tremor (ET) has traditionally been regarded as a pure motor disorder. However, emerging evidence suggests associations with non-motor symptoms, including cognitive impairment, psychiatric disturbances, and sleep abnormalities. REM Sleep Behavior Disorder (RBD) is a known prodromal feature of alpha-synucleinopathies such as Parkinson’s disease (PD). The relationship between ET and RBD remains unclear, with previous studies reporting variable prevalence rates.

**Objective:**

This study aimed to determine the prevalence of probable RBD (pRBD) among patients with ET using the REM Sleep Behavior Disorder Single-Question Screen (RBD1Q).

**Methods:**

In this single-center, cross-sectional study, we identified ET patients from the Parkinson’s Disease Center and Movement Disorders Clinic at Baylor College of Medicine. Eligible patients were contacted and screened for pRBD using the RBD1Q. A small cohort of healthy controls, including spouses, was also screened. Demographic and clinical characteristics were compared between ET patients with and without pRBD.

**Results:**

Among 245 ET patients, 21.2% screened positive for pRBD. There were no significant differences in age, age at tremor onset, or tremor duration between patients with and without pRBD. There was a significantly higher percentage of males among ET patients with pRBD (63.5% vs. 48.7%, p = 0.045).

**Discussion:**

In this study, pRBD screen positivity was relatively more common in ET patients than historical controls. These findings suggest that sleep-related symptoms may be relevant in ET and support further study with polysomnographic confirmation of RBD and longitudinal follow-up of these symptoms in ET patients.

## Introduction

Essential tremor (ET) is characterized by a chronic action tremor affecting both upper limbs, potentially extending to other body sites [1,2,3]. Globally, prevalence rates of ET in adults range from 0.4% to 6%, impacting approximately 1% of the general population and 4–5% of individuals aged over 65 years [4,5,6].

The conventional perspective depicting ET as an isolated tremor disorder has been contested [3,7,8]. Recognition of frequent “soft” neurologic signs that accompany ET, such as questionable dystonia or ataxia, led to the development of the ET plus diagnosis [1]. Emerging evidence suggests a potential association between ET and various non-motor symptoms (NMS), including cognitive impairment, anxiety, depression, and olfactory dysfunction [9,10,11,12,13]. ET has been raised as a potential risk factor for developing Parkinson’s disease (PD) [14,15], and one clinic-based phenotyping study reported 26.0% of ET patients in one series of patients in a movement disorders clinic had concurrent PD [14]. Reports indicate that individuals with ET may be four to five times more likely to develop PD than the general population, and there may be an association between ET and alpha-synucleinopathies [16]. However, definitive evidence of a link remains lacking.

Rapid eye movement (REM) sleep behavior disorder (RBD) is a sleep condition characterized by abnormal movements during REM sleep, often involving the enactment of dream content [17]. Stefani et al found the RBD questionnaires have low specificity and low positive predictive value and should not be used as a standalone tool for the diagnosis of RBD [18]. However, in a meta-analysis of 19 studies, the prevalence of RBD diagnosed by polysomnogram (PSG) was reported as 0.68%, and the prevalence of probable RBD (pRBD) as determined by a variety of scales, including the RBD Screening Questionnaire (RBDSQ) and REM Sleep Behavior Disorder Single Question Screen (RBD1Q) was reported as 5.65% in the general population, with no significant gender difference [19,20]. They reported that prevalence of pRBD for studies using RBD1Q (*n* = 4) was 4.23% (95% CI 1.26–8.82; *I*^2^ = 99%) [19]. Many studies have established a link between RBD and alpha-synucleinopathies, including PD, dementia with Lewy bodies (DLB), and multiple system atrophy (MSA). The presence of PSG-confirmed RBD is a powerful predictor of the development of eventual alpha-synucleinopathies [21].

Given the high association between RBD and PD, the prevalence of RBD in ET patients may further elucidate the risk for PD in ET patients. Studies suggest that RBD is relatively common in patients with ET. However, prevalence estimates vary (11% to 33%) depending on diagnostic criteria and specific population samples. Many studies have fewer than 55 patients and lack controlled groups [17,22,23]. Sample size in these studies has been smaller due to utilizing PSG confirmation of the diagnosis. However, larger sample sizes may be assessed for pRBD by screening questionnaires, including the RBD1Q, which demonstrated a 93.8% sensitivity and 87.2% specificity in diagnosing RBD compared to a PSG gold standard [24].

This study aims to determine the prevalence of pRBD among approximately 250 patients diagnosed with ET utilizing the RBD1Q.

## Methods

This single-center, cross-sectional study examined patients from the Parkinson’s Disease Center and Movement Disorders Clinic (PDCMDC) at Baylor College of Medicine diagnosed with ET. Patients with a diagnosis of ET seen between 1/1/2018 to 2/13/2024 were identified using the electronic medical record (Epic). Patients’ clinical files were reviewed to confirm their diagnosis, and eligible patients were contacted by phone to participate in the project. Spouses of the patients were also invited to participate as healthy controls and answer an additional screening question. Verbal informed consent was obtained from all subjects. This study was approved by the Institutional Review Board of Baylor College of Medicine, IRB approval number: H-55112.

Patients 18 years and older with a documented diagnosis of ET were eligible for the study. ET was diagnosed by a movement disorders neurologist involved in the clinical care of the patient. We divided ET patients into three subtypes according to their motor features: Pure ET, ET plus (as defined by the 2018 Movement Disorder Society (MDS) Consensus Statement on the Classification of Tremors), and ET-PD if meeting criteria for diagnosis of concurrent Parkinson’s disease [1]. Patients with ET-PD, other potential alpha-synucleinopathies such as dementia with Lewy bodies or multiple system atrophy, or other atypical parkinsonisms were not included. Patients with rest tremor or slight parkinsonian signs that did not meet criteria for PD were retained in the ET cohort and analyzed descriptively as phenotypic features rather than excluded as ET-PD.

Information collected from the chart included age at the time of onset of ET, duration of the disease, biological sex at birth, family history of ET, and co-morbidities.

Participating subjects, including healthy controls, were asked the RBD1Q, which consists of a single question, answered “yes” or “no” : “Have you ever been told, or suspected yourself, that you seem to ‘act out your dreams’ while asleep (for example, punching, flailing your arms in the air, making running movements, etc.)?”. Subjects who answered “yes” were classified as having pRBD. Healthy controls were additionally asked whether they have been diagnosed with PD, ET, dementia or ataxia to help screen their eligibility as a control subject.

### Statistical analysis

Continuous variables are expressed as mean ± standard deviation and were compared using the independent-samples t-test. Categorical variables are expressed as percentages, and comparisons were performed using the chi-squared test (Fisher’s exact test was used when chi-squared assumptions were not fulfilled). P < 0.05 was considered statistically significant. Statistical analysis was performed using the Statistical Package for Social Sciences (SPSS) 20.0 version software package.

### Data Availability

Data for this study are available with approval from the corresponding author and Baylor College of Medicine’s Institutional Review Board (IRB) on reasonable request.

## Results

A total of 245 subjects with ET were recruited. The mean age in this cross-sectional study was 67.01 ± 14.69 years old, and 52% of the cohort were male. Among the patients, 41.9% had pure ET, 32% dystonia, 17.5% rest tremor, 16.7% other slight parkinsonism (not meeting criteria for diagnosis of PD), 7.3% ataxia, and 4.1% cognitive impairment. These motor and cerebellar features were not mutually exclusive, and some patients had more than one phenotypic feature. Among the entire cohort, 21.2% screened positive for pRBD by the RBD1Q.

There were no significant differences of age, age at onset of ET, and duration of ET between the ET patients with and without pRBD (Table 1). The mean age was similar (67.82 ± 14.91 years in non-pRBD group vs. 63.90 ± 13.65 years in pRBD group, p = 0.089). The age at onset of tremor was not significantly earlier in either group (41.18 years in pRBD vs. 44.89 years in non-pRBD, p = 0.230), and tremor duration was also not significantly different (22.50 years in pRBD vs. 22.61 years in non-pRBD, p = 0.969). There was a significantly higher percentage of males in the pRBD group (63.5% vs 48.7%, p = 0.045 by Fisher’s Exact Test).

**Table 1:** Demographic and Clinical Characteristics of ET Patients With and Without Probable RBD.


	RBD	NON-RBD	P VALUE

Age (y)	63.90 ± 13.65	67.82 ± 14.91	0.089 ^a^

Age at Onset (y)	41.18 ± 19.22	44.89 ± 20.09	0.230 ^a^

Disease duration (y)	22.5 ± 16.22	22.61 ± 16.91	0.969 ^a^

Male	63.5%	48.7%	0.045 ^b^


Abbreviations: ET = essential tremor; RBD = REM sleep behavior disorder; y = years. Statistical tests: *a* = Independent samples t-test, *b* = Fisher’s exact test.

The prevalence of overlapping clinical features, such as rest tremor, parkinsonism, dystonia, and ataxia, was not significantly different among patients with or without pRBD (Table 2).

**Table 2:** Phenotypic Features of ET Patients With and Without Probable RBD.


	RBD	NON-RBD	P VALUE

Pure ET	51.9%	39.4%	0.681 ^a^

Dystonia	28.8%	33.2%	0.923 ^a^

Rest tremor	11.5%	18.7%	0.612 ^a^

Parkinsonism	15.4%	17.1%	0.519 ^a^

Family History	57.7%	53.7%	0.557 ^a^

Ataxia	1.9%	8.8%	0.110 ^a^

Cognitive impairment	3.9%	4.1%	0.982 ^a^


Abbreviations: ET = essential tremor; RBD = REM sleep behavior disorder. Statistical tests: *a* = chi-squared test; Fisher’s exact test was used when chi-squared assumptions were not fulfilled.

Only a small number of healthy controls (n = 30) were able to be recruited, among whom pRBD prevalence by the RBD1Q was 6.67%. Because our locally recruited control sample was small, the control comparison was interpreted cautiously and used only in an exploratory context. Accordingly, the main comparison in this study was made against the historical control prevalence reported by Postuma et al., rather than against our small locally recruited control group [24].

## Discussion

The prevalence of pRBD in the general population has been estimated to range from a 4.9–7.7% using a variety of scales, including the RBDSQ and RBD1Q [19,20,25,26]. In Postuma’s 2012 study defining the RBD1Q, RBD prevalence as defined by RBD1Q positive screening was found in 12.8% of control subjects. Our study utilizing the RBD1Q found a 21.2% prevalence of pRBD in a cohort of 245 ET patients, a 67% increase compared to the Postuma control group, suggesting that ET patients may be at higher risk of developing pRBD. However, because our study relied on questionnaire screening rather than polysomnography, these findings should be interpreted as a comparatively high prevalence of pRBD symptoms in this ET study rather than as confirmed RBD. As the locally recruited control sample was very small, we used the historical control data from Postuma et al. primarily for context rather than for direct statistical comparison.

Outside of a significantly higher percentage of males among ET subjects with pRBD, demographic and clinical features were similar between those with and without pRBD, including no significant differences in the prevalence of rest tremor or slight parkinsonism between groups [16,17,23,27,28,29,30,31,32]. These findings should be viewed as exploratory and hypothesis-generating rather than evidence of a definitive biological link between ET and alpha-synucleinopathies.

Historically, ET had been considered a movement disorder with pure motor manifestations, but emerging evidence suggests it is a heterogeneous condition. Rest tremor and subtle parkinsonian features not meeting criteria for Parkinson’s disease have been included in the ET spectrum under the ET plus diagnostic category [1]. A subset of patients display non-motor symptoms such as cognitive impairment, psychiatric disturbances, and sleep abnormalities [3,33]. As a higher prevalence of pRBD would suggest a higher risk of the eventual development of an alpha-synucleinopathies, our study may join others in supporting ET as a potential risk factor for PD [16,17,23,27,28,29,30,31,32,34,35,36,37]. However, particularly without PSG confirmation of RBD, our findings do not establish a direct relationship between ET and alpha-synucleinopathies. Future studies incorporating polysomnographic confirmation will be needed to clarify the clinical significance of pRBD in ET. Our cohort also included patients with additional neurologic signs, and these features should be interpreted as overlapping phenotypic characteristics rather than mutually exclusive categories. Accordingly, the current study should be viewed as describing a clinically heterogeneous ET cohort rather than a sample of pure ET only.

A significant finding of our study was the association between male sex and increased pRBD prevalence. Men exhibited a significantly higher prevalence (26.0%) vs. women (16.1%) (p = 0.04). The underlying mechanisms remain unclear, but a sex difference in RBD occurrence has been reported [38,39,40]. There is an association between male sex and RBD in PD patients as well [41,42,43]. However, we did not perform multivariable logistic regression analyses; therefore, the association between male sex and pRBD should be interpreted as unadjusted and hypothesis-generating rather than independent of age or other potential confounders.

The increased prevalence of pRBD in ET patients without Parkinson’s disease suggests the possibility of shared neurobiological pathways with alpha-synucleinopathies but it should not be interpreted as proof of a direct link. Screening for pRBD may serve as a method of identifying ET patients at risk for neurodegeneration. At the same time, the overlapping presence of dystonia, rest tremor, slight parkinsonism, and ataxia in this cohort limits the ability to attribute pRBD findings to isolated ET alone. Most patients with ET do not develop PD, but our study highlights the need for longitudinal follow-up of ET patients, ideally with evaluation for RBD by PSG and biomarker investigation, to further characterize these patients who may be at higher risk of developing PD.

Despite the strengths of our study including a larger sample size and a well-defined ET cohort, several limitations should be acknowledged. The RBD1Q is a highly sensitive but less specific tool for the diagnosis of RBD [24]. As Stefani showed in their study, the gold standard to confirm RBD is PSG [18]. Therefore, our prevalence estimate should be interpreted as questionnaire-based screen positivity for pRBD rather than PSG-confirmed RBD. Future studies would benefit from PSG confirmation for more accurate RBD diagnoses. Diagnosis of pRBD by self-reported questionnaire may be confounded by medications and co-morbidities. Depression and antidepressant use have been associated with pRBD in other ET cohorts [44]. A prospective longitudinal study to assess PD conversion rates among ET patients would be ideal, but also pragmatically challenging due to the follow-up length required. Another limitation of our study is the lack of a sufficiently large control group, although we are able to compare our data to historical controls utilizing the RBD1Q. The small local control sample is a limitation, and comparisons to the ET cohort should be interpreted as exploratory. Future studies should aim to recruit a larger, well-matched control group to strengthen findings. In addition, the phenotypic features reported here were not mutually exclusive, and a subgroup analysis of pure ET versus ET-plus would further strengthen future work.

Our study, one of the largest to date to examine pRBD prevalence in ET patients, found a significantly higher pRBD prevalence in ET patients utilizing the RBD1Q than has been reported in the general population. These results highlight the heterogeneity of ET and suggest that ET patients may have a higher risk of developing an alpha-synucleinopathies. There was a significantly increased proportion of males among ET subjects with pRBD. Future longitudinal studies of sleep-related symptoms in ET, ideally with PSG confirmation of RBD and examination of alpha-synuclein biomarkers are needed to clarify the relationship between ET, RBD, and alpha-synucleinopathies.
